# Plasma miRNA can detect colorectal cancer, but how early?

**DOI:** 10.1002/cam4.1398

**Published:** 2018-03-23

**Authors:** Maria L. Wikberg, Robin Myte, Richard Palmqvist, Bethany van Guelpen, Ingrid Ljuslinder

**Affiliations:** ^1^ Department of Medical Biosciences, Pathology Umeå University Umeå Sweden; ^2^ Department of Radiation Sciences, Oncology Umeå University Umeå Sweden

**Keywords:** Colorectal cancer, early detection, plasma miRNA

## Abstract

Colorectal cancer (CRC) is a major cause of deaths worldwide but has a good prognosis if detected early. The need for efficient, preferable non‐ or minimally invasive, inexpensive screening tools is therefore critical. We analyzed 12 miRNAs in pre‐ and postdiagnostic plasma samples to evaluate their potential as CRC screening markers. We used a unique study design with two overlapping cohorts, allowing analysis of pre‐ and postdiagnostic samples from 58 patients with CRC and matched healthy controls. Plasma concentrations of miR‐15b, ‐16, ‐18a, ‐19a, 21, ‐22, ‐25, ‐26a, ‐29c, ‐142‐5p, ‐150, and ‐192 were measured by semi‐quantitative real‐time PCR. Concentrations of miR‐18a, ‐21, ‐22, and ‐25 in plasma from patients with CRC were significantly altered compared to healthy controls. Combined as a multimarker panel, they detected CRC with an AUC of 0.93. Furthermore, levels of these three miRNAs also showed different levels in the prediagnostic case samples close to diagnosis. Only miR‐21‐levels were elevated several years before diagnosis. Plasma levels of miR‐18a, ‐21, ‐22, and ‐25 show promise as screening biomarkers for CRC. However, based on our unique analysis of prediagnostic and postdiagnostic samples from the same patients, we conclude that circulating miRNAs elevated at diagnosis may not automatically be suitable for CRC screening, if the increase occurs too close to clinical diagnosis.

## Introduction

The need for more efficient and practical tools for colorectal cancer (CRC) screening is well established as the disease is the fourth greatest cause of death worldwide, and the prognosis is greatly influenced by tumor stage at diagnosis 1. Hence, finding early‐stage CRC is of great importance, and therefore, several screening programs, such as fecal occult blood test, flexible colonoscopy, stool DNA test, computed tomography, and double‐contrast barium enema, are used. Moreover, there are many studies investigating the value of CEA (carcinoembryonic antigen) and ca‐19‐9 as blood sample‐based screening markers 2, 3. Unfortunately, these methods are quite unreliable, cost‐ineffective, and sometimes unpleasant for the patient, emphasizing unequivocally the need for novel markers identifying patients with undiagnosed CRC 4, 5.

Recent years have attributed major knowledge in the research area of microRNA (miRNA), a group of short 20–22 bp nucleotides, noncoding RNAs which play key roles in many biological processes, including cancer development 6, and miRNAs have been shown to be remarkably stable in both tissue samples as well as in plasma and serum 7, 8. One reason for that is that miRNAs interact with Argonaute2, which keeps it protected from nuclease activity, therefore, making them excellent markers for blood‐based detection of CRC 9, 10, 11.

A number of studies have indeed shown that several miRNAs are of interest when aiming to detect CRC 12, 13, 14, 15. Kanaan et al. 16 found a panel of eight circulating miRNAs (miR‐532‐3p, 331, ‐195, ‐17, ‐142‐3p, ‐15b, ‐532, and ‐652) that could distinguish CRC from controls (AUC 0.829), Wang et al. 17 identified miR‐409‐3p, ‐7, and ‐93 as panel that discriminated CRC from healthy persons (AUC 0.897), Wang et al. 18 showed that miR‐601 and ‐760 separated CRC from normal controls (AUC 0.792), and Vychytilova‐Faltejskova et al. 19 distinguished patients with colon cancer from healthy donors using the miRNA‐signature miR‐23a‐3p, ‐27a‐3p, 142‐5p, and ‐376c‐3p (AUC 0.922). Even though these studies show the potential of miRNAs as markers for detection of CRC, the difficulties finding a robust panel are still evident and in none of the studies, prediagnostic samples were available as comparison of miRNA levels.

In this study, the aim was to detect changes in plasma levels of selected miRNAs long before the diagnosis of the disease. To achieve this, we needed to identify a panel of circulating miRNAs discriminating CRC from healthy controls with high sensitivity and specificity. Evidentially, it is of great interest to detect a CRC by a simple blood test before the patient even has developed symptoms, but it would also be a major advantage to find reliable data indicating which individuals have an increased risk of developing CRC later in life, something that has been the target of a vast number of studies in the last decade 20, 21. Our target questions were firstly to investigate the time frame of changes in miRNA plasma levels in patients and secondly to evaluate the possibility of diagnosing a patient with CRC with a blood sample.

## Material and Methods

### Study cohorts and sample collection

**Figure 1:**
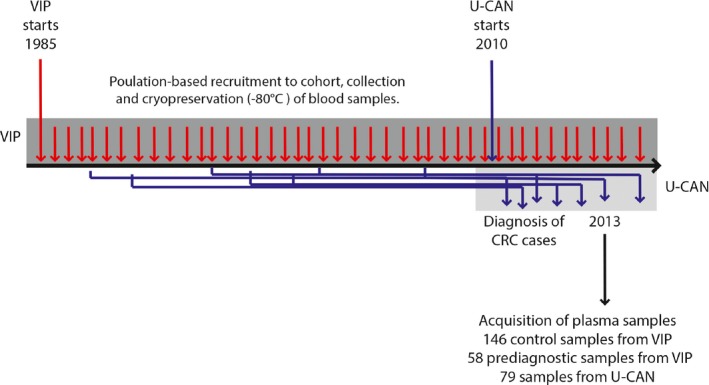
Study design. Plasma samples were collected for 67 CRC cases from the U‐CAN cohort. Plasma samples for double‐matched referent subjects were available from the VIP cohort, making it possible to analyze an increase in specific miRNA in patients with cancer. Additionally, prediagnostic plasma samples from the patients (up tp 21.6 years before diagnosis) were collected from the VIP cohort and analyzed for miRNAs, giving information if specific miRNAs can be detected in plasma early in cancer development.

The design of this study is unique. We accessed a large, population‐based cohort with prediagnostic samples from a large number of people who later developed CRC, as well as a clinical CRC biobank with postdiagnostic samples, to identify patients with CRC from whom both prediagnostic and diagnostic blood samples were available. The study design is shown in Figure 1.

Using the essential complete Cancer Registry of Northern Sweden, we identified 79 consecutive patients from the Uppsala‐Umeå Comprehensive Cancer Consortium (U‐CAN). Since 2010, all newly diagnosed patients with CRC in the Västerbotten County have been invited to the U‐CAN study. Blood samples and patient data are collected at diagnosis, but before surgery, as well as before, during, and after oncological therapy. The patients included in this study were diagnosed between 5 January 2010, and 24 February 2012, and included stage I‐IV patients with colorectal cancer. Two patients with previous history of CRC were excluded, six patients were excluded due to insufficient RNA quality, and four patients were found to have samples collected during ongoing chemotherapy leaving 67 patients for final analysis.

Of the 67 patients included in the study, 58 had donated prediagnostic blood samples to the population‐based Västerbotten Intervention Program (VIP). In VIP, established in 1985, all residents of the Västerbotten County, Sweden, are invited to participate in a health survey upon turning 30 (years, 1990–1996), 40, 50, and 60 years of age, comprising a medical examination and laboratory tests, donation of a fasting blood sample for future research, and completion of an extensive participant‐administered lifestyle and health questionnaire. The prediagnostic samples from the CRC cases were collected between 21.6 and 0.82 years before diagnosis.

For each CRC case in U‐CAN, two controls were selected from the VIP cohort, matched by age (±1 year), sex, and year of the diagnostic blood sample collection (U‐CAN). The study protocol and data handling procedures were approved by the regional ethical review board at Umeå University, Umeå, Sweden (dnr 2011‐292‐31‐M). None of the controls were diagnosed with CRC in the 5‐year period following the diagnosis of their matched case. All participants gave a written informed consent at the time of recruitment to the VIP and U‐CAN cohorts for all collection for research purposes. We attest that we have obtained appropriate permissions and paid any required fees for use of copyright‐protected materials. In both cohorts, the blood samples were collected in EDTA sample tubes, separated into plasma, buffy coat, and erythrocyte fractions, aliquoted and cryopreserved at −80°C at a central facility. Patient records were used to verify clinical and pathological data.

### miRNA analysis by semi‐quantitative real‐time polymerase chain reaction

Total RNA was extracted from 100 *μ*L plasma using the NucleoSpin miRNA plasma kit (Macherey Nagel, Düren, Germany) following the manufacturer's instructions. To monitor RNA recovery and reverse transcriptase efficiency, a synthetic miRNA was added after denaturation. This spike‐in miRNA, a *C.elegans* miR‐39 miRNA mimic (Qiagen, Chatsworth, CA), permits normalization between samples, which can control for varying RNA purification yields and amplification efficiency.

cDNA, synthesized using the miScript II RT kit (Qiagen) following the manufacturer's instructions, was used as template for real‐time PCR with the miScript SYBR Green PCR kit together with the miRNA‐specific assays (Qiagen). The reactions were performed in duplicates on a Taqman 7900HT (Applied Biosystems, Foster City, CA) using miRNA‐specific primers (Qiagen). The amplification profile started with denaturation at 95°C for 15 min, followed by 40 cycles of denaturation at 94°C for 15 sec, annealing at 55°C 30 sec, and extension at 70°C for 30 sec. The quantitation cycle (Cq) was calculated using the SDS 2.4 software (Applied Biosystems). Reliable and reproducible measurement of circulating miRNA is hampered by the fact that no known endogenous miRNA can be used for reliable normalization, and the amount of total RNA purified from plasma is so small that it is not readily quantified by UV‐absorbance measurements. In the absence of absolute determination of miRNAs, we standardized our miRNA amounts based on fixed plasma sample volumes and used spiked‐in synthetic nonmammalian miRNA to account for technical variation. The average Cq values of each miRNA were subtracted from the average Cq values of *C.elegans* miR‐39 for that particular sample, yielding a –ΔCq value. The –ΔCq values were further normalized by the average of Cq values for the whole data set. The normalized –ΔΔCq values were further used in the 2–ΔΔCq method 22, to obtain a relative value for each sample 23.

Six cancer plasma samples were lacking a proper RNA purification and were excluded from the study, leaving 67 for further analysis.

### Statistical analysis

The plasma miRNA levels were log10‐transformed in all statistical analyses. We evaluated differences in plasma miRNA concentration between cases and controls, and between the prediagnostic and diagnostic case samples, using Wilcoxon's rank sum test. *P*‐values were corrected for multiple testing with the Bonferroni method.

Associations between miRNAs were estimated with Spearman's correlations. Associations between miRNA and clinical characteristics were estimated by modeling z‐transformed miRNA levels in linear regression models adjusting for age and sex. Contribution to miRNA variance for each clinical variable was tested by analysis of variance.

The diagnostic performance of each miRNA was evaluated using logistic regression to construct a prediction model. To create a multimarker panel, the miRNAs with Bonferroni‐corrected significant differences between cases and controls were included in a logistic regression model. The predicted probabilities from these models were then used to estimate area under the ROC‐curve (AUC), a measurement of prediction accuracy. To adjust for potential overestimation of prediction accuracy, we used the .632+ bootstrap method (based on 1000 bootstrap samples) to estimate AUC and sensitivities at cut offs yielding 80% and 90% specificity. We further estimated stage‐specific AUCs (stage I/II or III/IV) and tested for differences using the Delong test.

All analyses were made in R version 3.2.2. All tests were two‐sided, and *P*‐values below 0.05 were considered significant.

## Results

### Characteristics of the study population

Table 1 presents characteristics of the CRC patient group. Men and women were equally represented: colon cancer accounted for 63%, and rectal cancer for 37% of the cases. Some 21 patients (31%) had metastasized disease at diagnosis and collection of the diagnostic blood sampling.

**Table 1:** Baseline and clinical characteristics

Variable	Cases (%)	Controls (%)
Sex		
Men	33 (49)	66 (49)
Women	34 (51)	68 (51)
Age		
<60	17 (25)	34 (25)
60–69	27 (40)	54 (40)
≥70	23 (34)	46 (34)
Tumor site		
Colon	42 (63)	
Rectum	25 (37)	
TNM stage		
I	10 (15)	
II	21 (31)	
III	15 (22)	
IV	21 (31)	

### miRNA concentrations in plasma samples from patients with CRC and healthy controls

Plasma levels of 12 selected miRNAs (miR‐15b, ‐16, ‐18a, ‐19a, 21, ‐22, ‐25, ‐26a, ‐29c, ‐142‐5p, ‐150, and ‐192) were analyzed by quantitative PCR on plasma samples from patients with CRC and healthy controls. Three major clusters of positively correlated miRNAs were found as follows: cluster 1 (miR‐22, miR‐25, miR‐26a, miR‐29c, and miR‐142‐5p, miR‐192), cluster 2 (miR‐150), and cluster 4 (miR‐15b, miR‐16, miR‐18a, miR‐19a, and miR‐21, Fig. S1).

Plasma concentrations of miR‐21, ‐22, ‐18a, and ‐25 differed significantly between patients with CRC and controls after adjustment for multiple testing (Table 2).

**Table 2:** Diagnostic performance of miRNAs for colorectal cancer detection

	Median log10‐expression		*P* a	Adjusted *P* b	Apparent AUC	.632+ AUC	.632+ sensitivity	
	Controls	CRC cases					At 80% specificity	At 90% specificity
miR.21	−8.0	−6.8	<0.001	<0.001	0.84	0.84	78	62
miR.25	−8.9	−8.5	<0.001	<0.001	0.65	0.63	46	31
miR.18a	−10.2	−9.8	0.003	0.036	0.59	0.56	29	12
miR.22	−9.7	−10.2	0.004	0.046	0.59	0.55	26	12
miR.15b	−8.7	−8.4	0.005	0.054	0.58	0.57	28	20
miR.142.5p	−10.1	−9.7	0.015	0.176	0.57	0.54	22	13
miR.29c	−9.7	−9.6	0.116	1.392	0.53	0.5	22	13
miR.19a	−9.3	−9.1	0.595	7.144	0.49	0.45	14	6
miR.16	−8.4	−8.4	0.541	6.486	0.49	0.44	14	7
miR.192	−10.3	−10.4	0.555	6.658	0.49	0.46	19	9
miR.26a	−7.8	−8.0	0.806	9.674	0.48	0.43	17	8
miR.150	−9.0	−9.0	0.752	9.029	0.46	0.43	15	7

a Wilcoxon signed‐rank test for difference in miRNA expression distribution between CRC cases and controls.

b The *P*‐value was adjusted for multiple testing by the Bonferroni method by multiplying the nominal *P*‐value with 12 (the number of miRNAs analyzed).

miR‐21 had the best diagnostic performance, with .632+ AUC of 0.84. When the cutoff value was set to yield 80% specificity, the .632+ adjusted sensitivity was 78%. Most miRNAs (10 of the 12 assessed) had higher adjusted AUCs for detecting lower stage CRC compared to more advanced CRC although no difference was significant in the Delong test (Table 3).

**Table 3:** Stage‐specific diagnostic performance of miRNAs for colorectal cancer detection

	Stage I & II Apparent AUC	Stage I & II .632+ AUC	Stage III & IV Apparent AUC	Stage III & IV .632+ AUC	*P* a
miR.21	0.88	0.87	0.80	0.79	0.264
miR.25	0.65	0.62	0.65	0.62	0.822
miR.15b	0.63	0.60	0.54	0.5	0.383
miR.22	0.65	0.59	0.53	0.45	0.209
miR.18a	0.65	0.58	0.55	0.49	0.345
miR.142.5p	0.59	0.54	0.54	0.49	0.737
miR.16	0.56	0.49	0.48	0.41	0.196
miR.29c	0.52	0.48	0.54	0.47	0.625
miR.19a	0.54	0.47	0.42	0.4	0.350
miR.150	0.49	0.43	0.49	0.41	0.599
miR.192	0.48	0.42	0.51	0.45	0.512
miR.26a	0.49	0.42	0.47	0.41	0.922

a Delong test for differences in AUCs between early and advanced stage colorectal cancer.

Using a multimarker prediction model, including the four miRNAs with significantly different levels in cases and controls (miR‐21, ‐22, ‐18a, and ‐25), a .632+ adjusted AUC of 0.93 was achieved, and the sensitivity at cutoff values yielding 80% and 90% specificities was 81% and 67%, respectively (Table 4).

**Table 4:** Diagnostic performance of a multimarker logistic regression prediction model including miR‐21, miR‐25, miR‐18a, and miR‐22

	632+ AUC	632+ sensitivity	
		At 80% specificity (%)	At 90% specificity (%)
All CRC	0.93	81	67
CRC stage I & II	0.92	88	73
CRC stage III & IV	0.85	68	57

Including all miRNAs in the multimarker model did not markedly improve prediction accuracy (data not shown). For the multimarker panel, the AUC for stage I and II CRC was 0.92, and the sensitivity at cutoff values yielding 80% and 90% specificities was 88% and 73%, respectively. For stage III and IV, the models were slightly less accurate, with an adjusted AUC of 0.85, and sensitivities at cutoff values yielding 80% and 90% specificities of 68% and 57%.

### Plasma levels of miRNA in relation to clinicopathological factors

Concentrations of miRNA in diagnostic plasma samples in relation to clinicopathological traits of the patients with CRC are presented in Table S1. Patients with rectal cancer had higher plasma levels of miR‐29c, compared to patients with colon cancer. Patients with CRC with detected distant metastases at blood sampling had significantly lower levels of circulating miR‐15b and ‐19a. Patients with higher T‐stage had significantly lower levels of miR‐150, ‐142‐5p, and ‐29c and higher levels of miR‐25 and ‐26a. In general, although, no clear pattern could be found to distinguishing separate clinicopathological groups.

### miRNA levels in prediagnostic plasma samples

Our unique study design, where we have access to two partly overlapping cohorts, allows us to analyze archival plasma samples from cancer patients years prior to their cancer diagnosis. Consequently, this enables us to evaluate whether specific miRNAs may be good biomarkers for risk prediction or early detection of CRC.

Of the miRNAs with significantly different concentrations between cases and controls using diagnostic samples, all except miR‐18a also differed when prediagnostic case samples were used. (*P* < 0.001 to 0.004, Fig. S2). Of these, miR‐21 concentrations were also significantly higher in the diagnostic versus prediagnostic case samples (*P* < 0.001). The remaining three miRNAs showed no clear temporal change in plasma concentrations among cases (*P* = 0.10 for miR‐25, *P* = 0.39 for miR‐18a, and *P* = 0.05 for miR‐22).

### Increase in circulating miRNA levels with time before diagnosis

**Figure 2:**
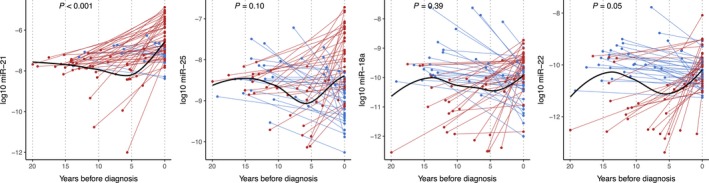
Plot of miR‐25, miR‐18a, and miR‐22 expression in prediagnostic and diagnostic blood samples from CRC cases by time before diagnosis. The connected lines are drawn between paired samples from each patient. Red lines indicate an increase and blue lines indicate a decrease in plasma miRNA concentrations from the prediagnostic to diagnostic sample. The black smooth line was estimated on all values using locally weighted scatterplot smoothing (loess). *P*‐values are from paired Wilcoxon signed‐rank test of differences between prediagnostic and diagnostic miRNA expressions.

As an important part of the study, we aimed to visualize when in time the plasma levels of miR‐21, ‐22, ‐18a, and ‐25 increased in prediagnostic samples, before diagnosis. Plasma concentrations of these miRNAs up to 20 years prior to diagnosis are presented in detail in Figure 2 (diagnosis denoted as year 0). All but 12 patients had an increase in miR‐21 levels from the prediagnostic to the diagnostic samples, which appeared to occur mainly during 3 years leading up to CRC diagnosis. Based on visual inspection of the figure, miR‐22 displayed a gradual, constant decrease in plasma concentrations during the prediagnostic period, with the exception of a subgroup of cases that, in contrast, showed a rather steep increase, particularly in the last few years before diagnosis. For miR‐25 and miR‐18a, no obvious pattern was apparent, although both steep increases and decreases over the prediagnostic period were common.

## Discussion

In this study, plasma concentrations of miR‐21, ‐25, ‐18a, and ‐22 differed between patients with CRC and matched controls. These differences were present even when prediagnostic samples from the same cases, collected up to 20 years prior to CRC diagnosis, were used. Furthermore, for miR‐21, a temporal effect was observed, with levels increasing over approximately 3 years leading up to CRC diagnosis. A multimarker panel based on miR‐21, ‐25, ‐18a, and ‐22 had diagnostic value for CRC yielding an AUC of 0.93, with 67% sensitivity at 90% specificity. The AUC for stage I and II CRC was 0.92, suggesting a potential use for early detection of CRC. Taken together, our findings, based on prediagnostic and postdiagnostic blood samples from the same group of patients, add important support to the potential of miRNA to improve current CRC risk prediction and screening strategies.

Research of miRNA as biomarkers of disease spans many areas of medicine from diabetes to gynecological and oral cancer 24, 25, 26, for example. However, few, if any, studies have the same unique design as reported in this study. Our archived samples were analyzed for a disease occurring several years after collecting the primary blood sample, investigating whether miRNA truly could be used to identify people who could benefit from earlier or more frequent colonoscopy screening.

The candidate miRNAs analyzed in this study were all chosen based on previously reported associations with CRC. miR‐21 is probably the most well‐documented miRNA in cancer research 27, 28, 29, 30, 31 and has previously been found to act as a marker for CRC in plasma and serum 32, 33, 34. Our findings provide additional evidence that miR‐21 is a valid tumor marker for CRC. Used alone, it resulted in an AUC of 0.84 and all but 12 patients with prediagnostic samples showed an increase in plasma miR‐21 concentration from the prediagnostic to the diagnostic sample. Increasing levels of miR‐21 have previously been associated with an increased risk of recurrence with liver metastases 35. For miR‐21 analyzed in CRC tissue from the primary tumor, increasing levels have also been correlated with a worse prognosis 36.

In recent years, increasing attention has focused on miR‐22, with results showing decreased levels in CRC tissue. miR‐22 has also been suggested to have antitumor effects 37, 38. In our study, miR‐22 showed lower levels in both diagnostic and prediagnostic samples from CRC cases compared to controls, which is in line with these previous findings.

For miR‐25, we detected significant differences in plasma concentrations between CRC cases and controls, which were significant even for the prediagnostic case samples. Levels in cases showed no clear change during the prediagnostic period. miR‐25 may, therefore, be a marker for increased risk of developing CRC, rather than a marker of existing cancer. If so, it could be useful for risk prediction modeling, for personalized colonoscopy screening strategies, but not for early detection of CRC. In studies from other groups, miR‐25 has been demonstrated as a prognostic factor that correlates with invasion and metastasis by regulation of the expression of the tumor suppressors PTEN and E‐cadherin in CRC tissue 39, 40.

miR‐18a has been found to have increased plasma concentrations in patients with CRC compared to healthy controls, but when analyzed in patients with advanced colorectal adenomas, the findings have not been concordant 41, 42, 43. Our results indicate that miR‐18a indeed is a marker for CRC, but the levels during the prediagnostic period show a too weak increase to be reliable as a screening marker.

Given our unique study design, the sample size was relatively large. However, the statistical power was limited. Also, one‐third of the cohort consisted of stage IV patients, which further limited the statistical power for analysis of early‐stage CRC. Although we detected statistically significant intraindividual changes in miRNA levels from the prediagnostic period to diagnosis, the cohort was too small to establish accurate cutoff levels for clinical use. The results presented in this study must therefore firstly be considered hypothesis generating, and the data will, accordingly, be validated in larger cohorts. The prediagnostic samples were collected up to 21.6 years prior to the diagnosis of the cases, which is a major strength of the study given the slow development of CRC, taking up to 2 decades to become an invasive tumor. The variable time to diagnosis reflects our study design, linking a large population‐based cohort with a clinical biobank, to obtain pre‐ and postdiagnostic samples from the same set of patients. This is a very rare opportunity, so rather than limiting the inclusion criteria to cases with prediagnostic samples within a certain time period prior to diagnosis, we chose to present the results for all available prediagnostic samples and include the issue of time in the analyses and interpretation. Degradation during storage could be an issue to contend with, but sample‐handling and storage conditions were of high quality, and data suggest that miRNAs are very stable over time 7, 8, 9, 10.

It is well established that miRNAs are stable molecules, shown to be expressed in varying levels in CRC, and could hypothetically be useful as a blood‐based tool for cancer detection. Several groups have analyzed levels of various circulating miRNAs, and many studies demonstrate promising results for the use of miRNA as a tool in the detection of CRC. However, to our knowledge, no previous investigation has included paired pre‐ and postdiagnostic samples from the same cases, probably due to the scarcity of this type of resource worldwide. Our study thus makes an important contribution to the field, demonstrating clearly that a high performance of miRNA markers in studies using postdiagnostic samples will not necessarily translate into clinical usefulness in a screening setting. Indeed, prediagnostic samples are critical to distinguish between biomarkers of risk, useful for risk prediction modeling, and biomarkers of disease, useful for disease screening.

In conclusion, this study, based on a unique analysis of paired prediagnostic and postdiagnostic samples from the same patients, offers novel insight into the potential of miRNAs as a tool for early detection of CRC. A multimarker panel based on miR‐21, ‐25, ‐18a, and ‐22 had diagnostic value for CRC, but only miR‐21 concentrations showed a clear temporal increase during the 3 years prior to diagnosis. Thus, circulating miRNAs elevated at diagnosis may not automatically be suitable for CRC screening, if the increase occurs too close to clinical diagnosis. Studies on larger patient cohorts, including subgroup analyses by tumor stage, are planned by our research group, aiming to find the optimal miRNA panel for CRC screening.

## Conflict of Interest

None declared.

## Supporting information


**Figure S1.** Correlations between studied miRNAs were estimated with Spearman's correlation coefficient.

**Figure S2.** Levels of (A) miR‐21, (B) miR‐25, (C) miR‐18a, and (D) miR‐22 in CRC cases (diagnostic and prediagnostic samples) and controls.

**Table S1.** Associations between plasma miRNA concentrations and clinical characteristics in colorectal cancer cases (n=67).
