# Supplementary figures and images for: Plasma miRNA can detect colorectal cancer, but how early?
Source: Cancer Med. 2018 Mar 23;7(5):1697–705. doi: 10.1002/cam4.1398 (PMC5943420; doi:10.1002/cam4.1398)

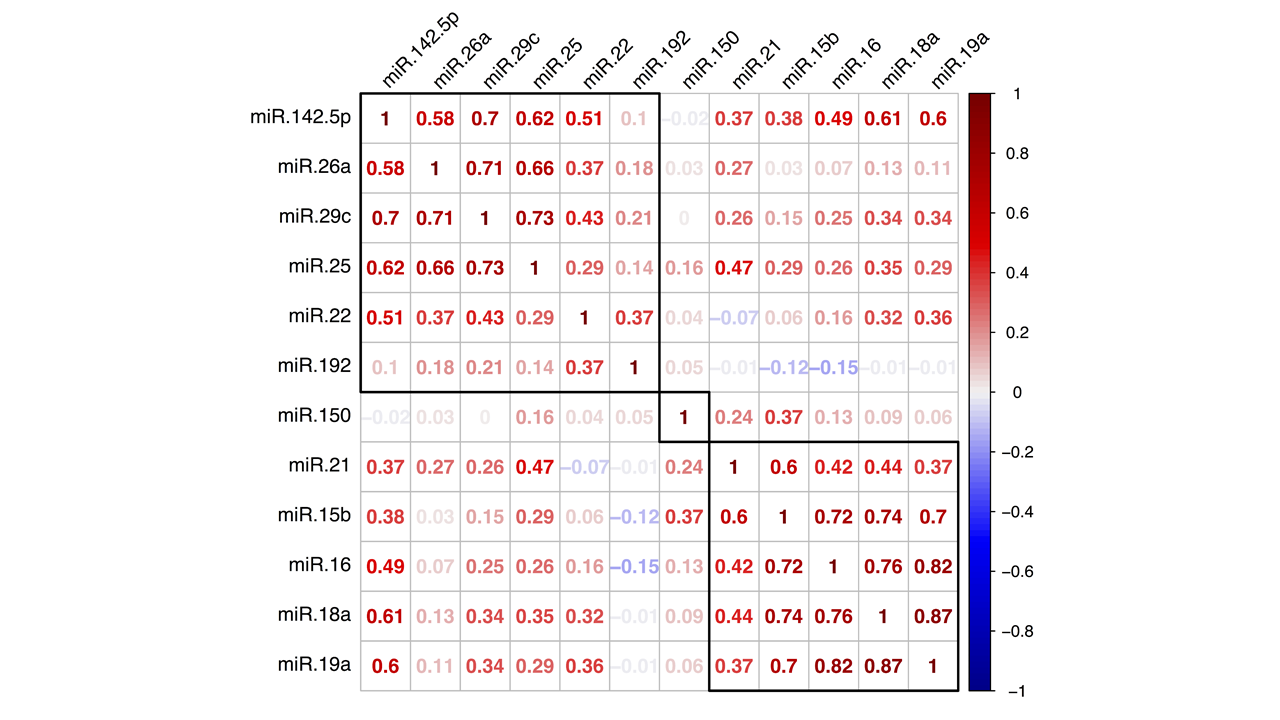

Supplement: Supplementary file 1 — Figure S1. Correlations between studied miRNAs were estimated with Spearman's correlation coefficient. [file CAM4-7-1697-s001.tif]

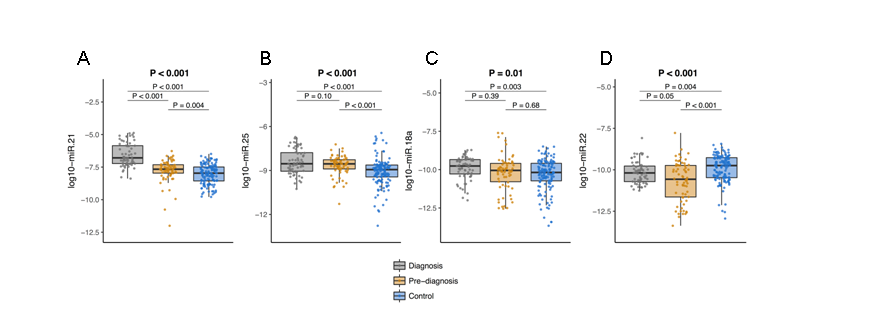

Supplement: Supplementary file 2 — Figure S2. Levels of (A) miR‐21, (B) miR‐25, (C) miR‐18a, and (D) miR‐22 in CRC cases (diagnostic and prediagnostic samples) and controls. [file CAM4-7-1697-s002.tif]
